# Transdermal rotigotine for the perioperative management of restless legs syndrome

**DOI:** 10.1186/1471-2377-12-106

**Published:** 2012-09-25

**Authors:** Birgit Högl, Wolfgang H Oertel, Erwin Schollmayer, Lars Bauer

**Affiliations:** 1 Department of Neurology, Innsbruck Medical University, Innsbruck, Austria; 2 Department of Neurology, Philipps University, Marburg, Germany; 3 UCB Biosciences GmbH, Monheim, Germany

## Abstract

**Background:**

Immobilisation, blood loss, sleep deficiency, and (concomitant) medications during perioperative periods might lead to acute exacerbation of symptoms in patients with the restless legs syndrome (RLS). Continuous transdermal delivery of the dopamine agonist rotigotine provides stable plasma levels over 24 h and may provide RLS patients with a feasible treatment option for perioperative situations. To assess the feasibility of use of rotigotine transdermal patch for the perioperative management of moderate to severe RLS, long-term data of an open-label extension of a rotigotine dose-finding study were retrospectively reviewed.

**Methods:**

The data of all 295 patients who had entered the 5-year study were screened independently by two reviewers for the occurrence of surgical interventions during the study period. The following data were included in this *post-hoc* analysis: patient age, sex, surgical intervention and outcome, duration of hospital stay, rotigotine maintenance dose at the time of surgery, rotigotine dose adjustment, and continuation/discontinuation of rotigotine treatment. All parameters were analysed descriptively. No pre-specified efficacy assessments (e.g. IRLS scores) were available for the perioperative period.

**Results:**

During the study period, 61 surgical interventions were reported for 52 patients (median age, 63 years; 67% female); the majority of patients (85%) had one surgical intervention. The mean rotigotine maintenance dose at time of surgery was 3.1 ± 1.1 mg/24 h. For most interventions (95%), rotigotine dosing regimens were maintained during the perioperative period. Administration was temporarily suspended in one patient and permanently discontinued in another two. The majority (96%) of the patients undergoing surgery remained in the study following the perioperative period and 30 of these patients (61%) completed the 5-year study.

**Conclusions:**

Although the data were obtained from a study which was not designed to assess rotigotine use in the perioperative setting, this *post-hoc* analysis suggests that treatment with rotigotine transdermal patch can be maintained during the perioperative period in the majority of patients and may allow for uninterrupted alleviation of RLS symptoms.

**Trial Registration:**

The 5-year rotigotine extension study is registered with ClinicalTrials.gov, identifier NCT00498186.

## Background

Restless legs syndrome (RLS), also known as Willis-Ekbom disease, is a common neurological disorder with substantial human and economic costs [1-3]. The disease remains underdiagnosed and also misdiagnosed in primary care [4] which recently prompted the proposal of diagnosis and treatment algorithms for primary care physicians/general practitioners by a task force sponsored by the European RLS study group [4]. RLS patients have an urge to move their legs (and sometimes arms and other body parts) during periods of rest and inactivity; this is usually accompanied or caused by unpleasant sensations in these limbs. The general circadian pattern (worsening in the evening and at night) can change as disease severity increases, and daytime symptoms develop [5].

One of the essential diagnostic criteria for RLS is the induction or exacerbation of symptoms by rest [6]; any form of immobilisation might therefore substantially increase symptom severity. Both leg discomfort and periodic leg movements indeed significantly worsened due to immobility in RLS patients but not in healthy controls [7]. Hospital stays for surgical interventions involving bed rest and possibly forced immobilisation during the postoperative period might therefore trigger or worsen symptoms in RLS patients. Additional factors potentially contributing to this exacerbation are illness- or pain-induced sleep deprivation, iron deficiency due to perioperative blood loss, and possibly the use of certain anaesthesia and concomitant medications such as neuroleptic agents, antiemetic agents with dopamine antagonistic properties, other dopamine antagonists, opioid antagonists, and some antihistamines and antidepressants [8,9]. New onset of RLS has also been reported following surgery with spinal anaesthesia. However, as lower mean corpuscular volume and haemoglobin pre-surgery were associated with new-onset RLS after surgery in this series, it cannot be excluded that iron deficiency accounted for new onset or exacerbation of RLS in that study [10]. Worsening of RLS symptoms might result in agitated patients with involuntary limb jerks during surgery, and general restlessness and major pain during recovery with ensuing postoperative complications and poor RLS symptom control for a prolonged period following surgery [11,12].

Current recommendations for perioperative RLS management suggest maintenance of RLS medication until just before surgery and resumption after surgery at full dose [13]. When using oral dopaminergic agents with a short half life, this temporary discontinuation might worsen RLS symptoms. Resuming full-dose treatment too quickly might also be problematic with dopamine agonists which require slow titration to avoid side effects. The dopamine agonist rotigotine, an efficacious and generally well tolerated treatment for RLS and Parkinson’s disease (PD) [14], might provide a suitable treatment alternative for RLS patients in the perioperative setting. Formulated in a transdermal patch, the continuous drug delivery generates stable rotigotine plasma concentrations over 24 h with once-daily application [15]. Administration of rotigotine transdermal patch may thus permit continuous alleviation of RLS symptoms in the perioperative setting. Previous studies considered rotigotine transdermal patch a feasible alternative for perioperative PD management [16,17]. Treatment was associated with good control of PD symptoms, easy switching and re-switching of regular PD medication, and a high patient acceptance [16].

In order to assess rotigotine transdermal patch for the perioperative management of RLS, long-term data of a rotigotine 5-year study were retrospectively reviewed.

## Methods

**Figure 1:**
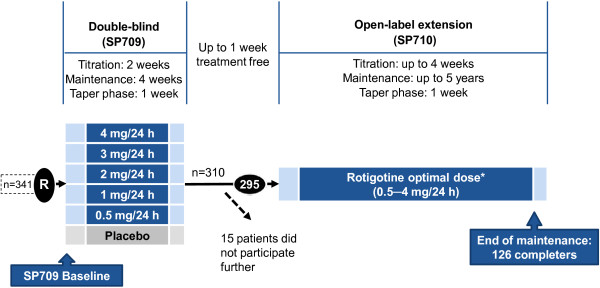
**Design of the 5-year open-label extension study with rotigotine transdermal patch in restless legs syndrome (adapted from Oertel et al. [**[19,21]**]). **Patients completing the double-blind study had the option of long-term treatment with their optimal dose of transdermal rotigotine (0.5-4 mg/24 h) in the open-label extension.

Data for this *post-hoc* analysis were obtained from a 5-year prospective open-label study of rotigotine treatment for moderate to severe RLS (SP710, NCT00498186, [18]), which is the extension of a 6-week randomised, double-blind, placebo-controlled rotigotine dose-finding study (SP709, NCT00243217) [19]. The study design is summarised in Figure 1. Patients eligible for participating in the dose-finding study were 18–75 years of age, had met the diagnosis of idiopathic RLS based on the four essential diagnostic criteria according to the International RLS Study Group (IRLSSG [6]), and had an IRLSSG severity rating scale (IRLS [20]) sum score ≥ 15 (= at least moderate RLS); complete inclusion/exclusion criteria are described elsewhere [19]. Study completers were given the option of long-term treatment with their optimal rotigotine dose (dose range 0.5-4 mg/24 h) provided they had no ongoing serious adverse events (AEs) suspected to be related to their randomly assigned treatment in the preceding dose-finding study. They were excluded for severe application site reactions or noncompliance in the preceding double-blind study. During the open-label extension, administration of concomitant treatments was kept to a minimum. Visits were scheduled at monthly intervals during the first year and at 3-monthly intervals thereafter. Both studies were performed according to the Declaration of Helsinki and Good Clinical Practice, and were approved by a central institutional review board in Germany (Kommission für Ethik in der ärztlichen Forschung im Fachbereich Humanmedizin der Philipps-Universität Marburg) and in Austria (Ethikkommission der Medizinischen Universität Innsbruck). In Spain, review and approval was provided by the local ethics committees of the Hospital Universitario La Princesa, Madrid, of the Hospital de la Ribera, Alzira/Valencia, and of the USP Institut Universitari Dexeus, Barcelona.

Written informed consent was obtained from all patients before participation.

The data of all 295 patients who had entered the open-label extension (mean age, 58.3 ± 10.1 years; median, 61 years; 66% female) were screened independently by two reviewers (ES, LB) for the occurrence of surgical interventions during the study period. They reviewed all clinical study report narratives of serious AEs and other significant AEs and crosschecked the obtained information against the Council for International Organisations of Medical Sciences (CIOMS) suspect adverse reaction report forms. In case of inconsistent data, the relevant narratives and CIOMS forms were re-examined by both reviewers in order to reach an agreement about the case.

The following data were extracted for all patients with a surgical intervention during the study period: patient age, sex, surgical intervention and outcome, duration of hospital stay, rotigotine maintenance dose at the time of surgery, rotigotine dose adjustment, and continuation/discontinuation of rotigotine treatment. All parameters were analysed descriptively. No pre-specified efficacy assessments (e.g. IRLS scores) were available for the perioperative period, i.e. before and after the surgical intervention.

## Results

### Surgical interventions

**Figure 2:**
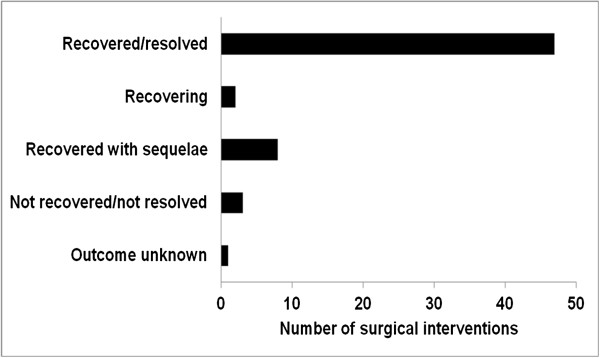
Reported outcome of surgical interventions.

During the 5 year study period, 61 surgical interventions were reported for 52 patients (17.6%). These patients had a mean age of 60.4 ± 11.6 years (median, 63 years) at study entry; 67.3% were female. Table 1 lists demographics and surgery details for each patient. Forty-four patients (84.6%) had one surgical intervention; seven patients had two procedures and one patient had three interventions. The duration of patients’ hospital stay ranged from 0 to 44 days. Surgeries consisted mainly of orthopaedic (45.9%), gynaecological/urological (14.8%), and cardiovascular (13.1%) procedures (Table 2). The most frequent surgery outcome was recovered/resolved (77.1%, Figure 2). According to the very detailed case descriptions, no complications, adverse events or other surgery, RLS or medication related issues were observed. A sub-analysis of the IRLS scores at time of surgery could not be conducted in this *post-hoc* analysis, because data were not available; the overall study population presented with a mean IRLS score of 27.8 ± 5.9 at baseline, indicating moderate to severe RLS [21]. Although eligibility criteria of the original study stipulated the consistent use of two combined effective contraception methods (including at least one barrier method) unless sexually abstinent, one patient became pregnant and had a planned abortion. The investigator considered the abortion as not related to trial medication. The subject had been exposed to the trial medication for 607 days and was withdrawn from the study 17 days prior to the abortion.

**Table 1:** **Characteristics of the patients included in the *post-hoc *analysis**

**Patient**	**Sex**	**Age**^**a**^**(years)**	**Surgical intervention**	**Concomitant medication prior to surgery**	**Surgery related concomitant medication**	**Rotigotine dose at time of surgery (mg/24 h)**	**Rotigotine dose following surgery (mg/24 h)**	**Outcome of surgery**
10306	Female	60	Bypass and tricuspidal reconstruction	Acetylsalicylic acid 100 mg/day, fluvastatin 80 mg/day, pantoprazole sodium 40 mg/day, telmisartan 1 mg/day.	No concomitant medications recorded	4	4	recovered/resolved
10311	Female	30	Abortion	No concomitant medications recorded	No concomitant medications recorded	2	Discontinued 17 days prior to surgery	recovered/resolved
10402	Female	54	Right knee replacement	Zopiclone 3.75 mg/as needed	Ibuprofen 1800 mg/day after surgery	4	4	recovered/resolved with sequelae
10404	Female	63	Surgery for dislocated fracture of left distal radius	Levothyroxine 100 μg/day	Furosemide 20 mg/day, acemetacin 60 mg- 180 mg/day, paracetamol 4 g/day	2	2	recovered/resolved
10429	Female	58	Right hip replacement	Estradiol valerate twice weekly (unknown dose), levothyroxine sodium 50 mg/day, siccaprotect 3 drops/day (unknown dose)	Indometacin 75 mg/day	3	3	not recovered/resolved (osteoarthritis was considered to be ongoing)
10702	Male	64	Cardiac bypass surgery Right shoulder surgery (osteoarthritis) Angioplasty (internal carotid artery)	*Cardiac bypass surgery*: fenofibrate 160 mg/day, serenoa repens extract 320 mg/day, Sidros 80.5 mg/day, tamsulosin 0.4 mg/day *Right shoulder surgery:* heparin-fraction, sodium salt (dose unknown), torasemide 10 mg/day, bisoprolol 5 mg/day, simvastatin 40 mg/day *Angioplasty:* no additional medication	*Cardiac bypass surgery*: acetylsalicylic acid 100 mg/day, torasemide 10 mg/day, bisoprolol 5 mg/day, simvastatin 40 mg/day *Right shoulder surgery: *Ultracet (1tab/as needed), esomeprazole 20 mg/day, diclofenac 100 mg/day *Angioplasty: *no additional medication	2	2	*Cardiac bypass surgery*: recovered/resolved *Right shoulder surgery: *recovered/resolved *Angioplasty:* recovered/resolved
10708	Female	62	Hip replacement	Diclofenac 150 mg/day, estradiol valerate 1 mg/day	No concomitant medications recorded	3	3	recovered/resolved
10713	Female	70	Thyroid surgery Hip replacement	*Thyroid surgery*: simvastatin 40 mg/day, carbamazepine 400 mg/day *Hip replacement: *simvastatin 40 mg/day. levothyroxine 75 mg/day	*Thyroid surgery*: no concomitant medications recorded *Hip replacement*: no concomitant medications recorded	4	4	*Thyroid surgery*: recovered/resolved *Hip replacement*: recovered/resolved
10801	Female	49	Surgery for left lower leg fracture	Calcium with vitamin D (dose unknown), risedronate sodium 35 mg/weekly, omeprazole 40 mg/day.	No concomitant medications recorded	3	3 (trial medication was temporarily suspended for the surgery and later resumed)	recovered/resolved
10806	Female	43	Hysterectomy	Timolol 1drop/day, domperidone 20 mg/day, acetylcysteine 600 mg/day	No concomitant medications recorded	4	4	recovered/resolved
10906	Female	70	Right knee replacement	Lisinopril 5 mg/day, acetylsalicylic acid 100 mg/day, trospium chloride 5 mg/day, ibuprofen 600 mg/every other day.	No concomitant medications recorded	4	4	resolved with sequelae
10907	Female	68	Hallux valgus surgery	Propranolol 50 mg/day, bisoprolol 50 mg/day, estriol 0.5 mg/biweekly	Heparin (dose unknown), ibuprofen 1800 mg/day	4	4	recovered/resolved with sequelae
10908	Female	44	Arthroscopy of right knee	No concomitant medication reported	Ibuprofen 600 mg/day	2	2	not recovered/not resolved
10914	Female	71	Right knee replacement Left knee replacement	*Right knee replacement*: bisoprolol 142.5 mg/day, ramipril 1.25 mg/day *Left knee replacement*: bisoprolol 142.5 mg/day, ramipril 1.25 mg/day	*Right knee replacement*: no concomitant medications recorded *Left knee replacement*: omeprazole 20 mg/day, enoxaparin 40 mg/day, tilidine hydrochloride 200 mg/day, indometacin 75 mg/day, metamizole 20 drops/as needed, gabapentin 900 mg/day	4	4	*Right knee replacement*: resolved with sequelae *Left knee replacement:* recovered/resolved with sequelae
10915	Female	53	Cholecystectomy	Opipramol 150 mg/day, pantoprazole sodium 20 mg/day	No concomitant medications recorded	0.5	0.5	recovered/resolved
11108	Female	49	Left shoulder surgery (impingement syndrome)	Estradiol valerat/norgestrel (administered in monthly cycle; 21 doses over 28 days)	No concomitant medications recorded	4	4	recovered/resolved
11110	Female	70	Hysterectomy	Theophylline 400 mg/day, ibuprofen 400 mg/day, fluticasone propionate/salmeterol xinafoate (dose unknown), cromoglicate sodium/reproterol hydrochloride (dose unknown), allopurinol 300 mg/day	No concomitant medications recorded	2	2	recovered/resolved
11112	Female	23	Submandibular cyst resection	No concomitant medications recorded	No concomitant medications recorded	2	2	recovered/resolved
11209	Male	47	Perianal abscess resection	Diltiazem 90 mg/day, pentaerithrityl tetranitrate 80 mg/day	Ibuprofen 1600 mg/day (discontinued because of an allergic reaction) thereafter tramadol 60 drops/day	3	3	recovered/resolved
11211	Male	62	Left knee replacement	Candesartan cilexetil (dose unknown)/day, celecoxib 100 mg/day, doxazosin mesilate (dose unknown)/day, dyazide 1tab/day, etoricoxib 60 mg/day, ibuprofen 600 mg/day, metoprolol succinate 95 mg/day, rofecoxib (dose unknown)/day	Heparin-fraction/sodium salt 2 mL /day, metamizole sodium 2000 mg/mL/day, tramadol hydrochloride 200 mg/day	4	4 (after surgery), discontinued 23 days after surgery owing to rehabilitation	recovered/resolved
11405	Female	64	Surgery for lumbar spinal cord stenosis Left shoulder surgery (frozen shoulder)	*Surgery for lumbar spinal cord stenosis: *no concomitant medications recorded *Left shoulder surgery: *cefuroxime 1000 mg/day.	*Surgery for lumbar spinal cord stenosis: *no concomitant medications recorded *Left shoulder surgery: *no concomitant medications recorded	4	4	*Surgery for lumbar spinal cord stenosis: *recovered/resolved *Left shoulder surgery: *recovered/resolved
11408	Female	63	Vein stripping (both legs)	Olmesartan medoxomil 20 mg/day	Initially alfetanil, mepivacaine, midazolam, propofol (doses unknown). Thereafter ibuprofen (dose unknown)	3	3	recovered/resolved
11419	Male	45	Right shoulder surgery	No concomitant medications recorded	Ibuprofen 400 mg/as needed	4	4	recovered/resolved
11428	Female	76	Right thumb surgery (arthrosis metacarpophalangeal)	Cyanocobalamin 1 mL/month, pantoprazole sodium 40 mg/day	No concomitant medications recorded	4	4	recovered/resolved
11430	Female	60	Hallux valgus surgery	No concomitant medications recorded	Diclofenac 50 mg/as needed	2	2	recovered/resolved
11501	Male	74	Hip replacement	Alprostadi cream 10 μg/day, candesartan cilexetil 4 mg/day, itraconazole solution (dose unknown)/day, nifedipine 10 mg/day	No concomitant medications recorded	4	4	recovered/resolved
11503	Female	63	Tendon repair (supraspinatus)	Captopril 17.5 mg/day, levothyroxine sodium 50 μg/day, simvastatin 20 mg/day plus unspecified pain medication	No concomitant medications recorded	4	4	recovered/resolved with sequelae
11601	Female	67	Right knee replacement	Acetylsalicylic acid 300 mg/day, bisoprolol (dose unknown), conjugated estrogens (dose unknown)	Ibuprofen 800 mg/as needed, cortisone (dose unknown)/as needed	4	4	recovered/resolved
11608	Male	76	Surgery for salivary gland adenoma	Enalapril maleate 10 mg/day, amiloride hydrochlorothiazide 5 mg/day, ramipril 5 mg/day	No concomitant medications recorded	4	4	recovered/resolved with sequelae
11609	Male	66	Coronary artery disease, stent implant	No concomitant medications recorded	Acetylsalicylic acid 100 mg/day and blood coagulation factors 75 mg/day for 5 ½ months, thereafter asasantin 1 capsule/day; bisoprolol 5 mg/day	4	4	recovered/resolved
11612	Male	71	Surgical treatment of lipoma	No information available	No information available	Discontinued in order to plan for surgical treatment of the lipoma	Discontinued	ongoing
11811	Male	64	Hand surgery (Dupuytren’s contracture)	Dermatologicals 1 mg/day cream, enalapril maleate 0.5 mg/day, hydrochlorothiazide and 0.5 mg/day heparin-fraction/sodium salt 2500 IU/day (day prior to surgery)	Heparin-fraction/sodium salt 2500 IU/day, ibuprofen 600 mg/as needed	3	3	recovered/resolved
11814	Male	43	Laparoscopic surgery (inguinal hernia)	Ibuprofen 800 mg as needed, levothyroxine sodium/potassium iodide 75 μg/day	No concomitant medications recorded	4	4	recovered/resolved
11901	Female	71	Surgical elevation of bladder Radius fracture surgery	*Surgical elevation of bladder: *ginkgo biloba extract 80 mg/day, acetylsalicylic acid 100 mg/day, calcium compounds 1500 mg/day, bisoprolol 5 mg/day, estrogen 0.6 mg/day, omeprazole 20 mg/day, diclofenac 75 mg/prn, hypericum perforatum 1350 mg/day, losartan 50 mg/day *Radius fracture surgery: *acetylsalicylic acid 100 mg/day, bisoprolol 10 mg/day, bromazepam 6 mg/as needed, calcium compounds 1500 mg/day, diclofenac 75 mg/as needed, dimethindene drops 4.5 mg/day, estrogen 0.6 mg/day, furosemide 40 mg/day, ginkgo biloba extract 80 mg/day, hypericum perforatum 1350 mg/day, losartan 50 mg/day, omeprazole 20 mg/day, prednicarbate cream (dose unknown). *Prior to surgery*: metamizole 1000 mg/day, tilidine 100 mg/day, enoxaparin sodium 0.4 mL/day (prophylaxis of thrombosis), cefuroxime 1.5 mg/day (inflammatory prevention), bupivacaine 10 mL, mepivacaine 40 mL (anaesthesia), clorazepate dipotassium 10 mg (tranquilizer) midazolam 6 mg (sedative)	*Surgical elevation of bladder: *enoxaparin 40 mg/day, magnesium 1500 mg/day, ethinyl estradiol 20 mg/day, promethazine 25 mg/day, midazolam 3.75 mg/day, sultamicillin 1125 mg/day. *Radius fracture surgery:* tilidine 100 mg/day	0.5	0.5	*Surgical elevation of bladder*: recovered/resolved *Radius fracture surgery*: recovered/resolved
11902	Female	66	Surgical elevation of bladder	Esomeprazole 20 mg/day, pantoprazole 20 mg/day, methotrexate 25 mg/week, prednisolone 5 mg/day, metoprolol 25 mg/day, calcium folinate 6.35 mg/week, leflunomide 20 mg/day, nitrofurantoin 50 mg/day, vitamin C + calcium (dose unknown)	Sulfamethoxazole (dose unknown)	4	4	recovered/resolved
12102	Female	59	Repair of incisional hernia	Diclofenac 75 mg/as needed, ibuprofen (unknown dose)/as needed, oestradiol/norethisterone acetate (unknown dose)/day, levothyroxine sodium 50 μg/day, omeprazole 20 mg/day	Certoparia sodium 9000 units/day, thereafter novaminsulfon 1 mL/day	3	3	recovered/resolved
12103	Female	56	Colon adenoma ablation	Norethisterone acetate (dose unknown), estradiol (dose unknown), acetylsalicylic acid 750 mg/as needed	No concomitant medications recorded	1	1	recovered/resolved
12208	Male	66	Appendectomy	Acarbose 150 mg/day, amlodipine 5 mg/day, clopidogrel sulfate 75 mg/day, fenofibrate 200 mg/day, ferrous sulfate 50 mg/day, glibenclamide 7 mg/day, lisinopril 5 mg/day, simvastatin 20 mg/day	No concomitant medications recorded	3	3	recovered/resolved
12307	Female	59	Right knee replacement Lumbar disk surgery	*Right knee replacement: *diclofenac 75 mg/day (plus physiotherapy) *Lumbar disk surgery*: ibuprofen 600 mg/as needed, thereafter paracetamol/codeine phosphate 1560 mg/day and metamizole sodium 1500 mg/day	*Right knee replacement: *diclofenac 150 mg/day, metamizole 1500 mg/day *Lumbar disk surgery*: oxycodone/naloxone 30 mg/day, tilidine drops (dose unknown)/as needed, fentanyl patch 50 μg/week	4	4	*Right knee replacement*: recovered/resolved with sequelae *Lumbar disk surgery*: recovering/resolving
12315	Male	46	Meniscus surgery	Acetylsalicylic acid 100 mg/day.	No concomitant medications recorded	3	3	recovered/resolved
12401	Male	66	Prostate resection	Bisoprolol hemifumarate 5 mg/day, flecainide acetate 100 mg/day, lansoprazole 15 mg/day, valsartan drops 160 mg/day	No concomitant medications recorded, no chemotherapy, no tumor markers	4	4	recovered/resolved
12603	Female	63	Vein stripping (both legs)	Biotin 2.5 mg/week, calcium 500 mg/day, ergocalciferol 0.025 mg/day, estradiol (dose unknown), zinc 25 mg/week, unspecified other urologicals, including antispasmodics. *Prior to surgery*: 40 mg/day heparin fraction/sodium salt for the prevention of thrombosis	No concomitant medications recorded, compression stockings recorded as therapy	2	2	recovered/resolved
12606	Male	63	Vein stripping (right leg)	Acetylsalicylic acid 300 mg/day, allopurinol 300 mg/day, bisoprolol 5 mg/day, insulin 48 IU/day, metformin 850 mg/day, ramipril 1 mg/day	No concomitant medications recorded	3	3	recovered/resolved
12701	Male	70	Pelvic bypass surgery	Acetylsalicylic acid 200 mg/day, nafti-ratiopharm retard 400 mg/day	No concomitant medications recorded	3	3	recovered/resolved with sequelae
13001	Female	72	Surgery for left orbital fracture	Mesalazine 1500 mg/day, amlodipine 5 mg/day	No concomitant medications recorded	4	4	recovered/resolved
13002	Female	49	Hysterectomy	No concomitant medications recorded	No concomitant medications recorded	4	4	recovered/resolved
13013	Female	75	Hip replacement (left) Hip replacement (right)	*Hip replacement (left) and hip replacement (right):*valsartan/hydrochlorothiazide (dose unknown), diclofenac 100 mg/as needed and 75 mg/as needed, diclofenac potassium 50 mg/as needed, lercanidipine 10 mg/day, metoprolol (dose unknown)	*Hip replacement (left) and hip replacement (right):* no concomitant medications recorded	0.5	0.5	*Hip replacement (left)*: recovered/resolved *Hip replacement (right): *recovered/resolved
13102	Female	63	Vertebral fusion (spondylolisthesis) Vertebral fusion (spondylolisthesis)	*Both surgeries*: lercanidipine 10 mg/day, pravastatin sodium 40 mg/day, ibuprofen 400 mg/as needed.	*Both surgeries*: no concomitant medications recorded	4	4	*Both surgeries*: recovered/resolved
14007	Male	66	Osteotomy of right distal tibia	Heparin-fraction/sodium salt 40 mg/day,	Heparin-fraction/sodium salt 40 mg/day, naproxen 1000 mg/day, pantoprazole 20 mg/day	3	3	recovered/resolved
17005	Female	47	Hysterectomy	Ibuprofen 600 mg as needed	No concomitant medications recorded	3	3	recovered/resolved
17007	Female	71	Hip prosthesis replacement	Chondroitin sulfate sodium 800 mg/day, glucosamine sulfate 1500 mg/day, ibuprofen 400 mg/as needed, paracetamol 600 mg/as needed	Acetylsalicylic acid 300 mg/day, enoxaparin 40 mg/day, alendronate sodium 70 mg/week, calcium 500 mg/day, ciprofloxacin 1500 mg/day, paracetamol 4 g/day, metamizole 150 mg/day, omeprazole 20 mg/day, thiamine nitrate/pyridoxine hydrochloride, clonazepam 2.5 mg/day (for RLS, immediately after surgery)	4	4	recovering/resolving
17301	Male	71	Bladder resection	Pentoxifylline 800 mg/day, tamsulosin 0.4 mg/day	No concomitant medications recorded	4	4	recovered/resolved

^a ^at start of open-label extension period.

**Table 2:** Surgical interventions during the 5-year rotigotine study

**Surgical intervention**	**Number**
Orthopaedics	28
Gynaecology/Urology	9
Cardiovascular	8
Trauma	6
Abdomen	5
Other	5

### Rotigotine dosing during the perioperative period

The rotigotine maintenance dose at the time of surgery is listed for each patient in Table 1. The mean dose was 3.1 ± 1.1 mg rotigotine/24 h (median, 3.5 mg/24 h). For the majority of interventions (n = 58, 95.1%), rotigotine dose regimens were maintained during the perioperative period. Administration was temporarily suspended in a 49-year old female patient for leg fracture surgery. Treatment was later resumed and the patient completed the study. Two patients permanently discontinued rotigotine treatment prior to surgical intervention; a 30-year old female patient stopped rotigotine administration 17 days before her surgery due to pregnancy (study withdrawal criterion) and a 71-year old male patient withdrew in order to prepare for upcoming lipoma surgery on his right lower leg. Tumor growth had been noticed by the patient prior to the start of the study.

### Study completion

In total, 50 (96.2%) of the patients undergoing surgery remained in the study following the perioperative period and 30 of these patients (61.2%) completed the 5-year study. The other 19 patients discontinued prematurely; reasons were lack of efficacy (6 patients), adverse events (4 patients, one case each of gambling, hallucinations, application site pruritus, and osteoarthritis), unsatisfactory compliance (3), major protocol violations (3), withdrawn consent (1) and others (2).

## Discussion

Bed rest, forced immobilisation, pain-induced sleep deprivation, iron depletion owing to intraoperative blood loss, and medications can all exacerbate RLS symptoms during the perioperative period [9]. Additionally, the temporary discontinuation of oral RLS medication before surgery and resumption at full dose postoperatively which is currently recommended for the perioperative RLS management [13] might worsen symptoms during surgery and lead to the occurrence of side effects when re-establishing the medication postoperatively. Administration of a medication such as rotigotine transdermal patch which provides stable plasma concentrations over 24 h with once-daily application may allow an uneventful continuous management of RLS symptoms in the perioperative setting.

To investigate this hypothesis, a retrospective analysis was carried out to obtain information about the perioperative management of RLS with rotigotine transdermal patch. Data from all patients undergoing surgery during the study period were extracted from the database of a 5-year open-label rotigotine study [18]. As can be expected from a database with a median age of 61 years, surgical interventions occurred frequently [22]: nearly one fifth of all patients underwent surgery during the 5-year study period, consisting mainly of orthopaedic, gynaecological/urological, and cardiovascular procedures. Treatment with rotigotine transdermal patch could be continued throughout the perioperative period in all but 3 of these patients without a change in rotigotine maintenance dose.

Rotigotine transdermal patch might be useful in the perioperative setting for the continuous alleviation of the usual RLS symptoms experienced by the patients. Additionally, the continuous drug delivery might counteract involuntary movements during surgery or during recovery triggered by bed rest and immobilisation, illness- or pain-induced sleep deprivation, iron depletion, (concomitant) medications, or certain anaesthetics. Several case reports have described the transient occurrence of RLS and periodic limb movements with epidural or spinal anaesthesia [10,23-28] which might worsen the symptoms already present in RLS patients and might lead to interference with surgical procedures and a prolonged recovery time.

This investigation was a retrospective analysis and not a prospective study providing efficacy data such as IRLS values for the perioperative period. It should also be noted that the original study from which these data were obtained was not designed to assess the use of rotigotine transdermal patch in the perioperative setting. To our knowledge, no pharmacokinetic studies investigating interactions with medications commonly used during surgery have been conducted for rotigotine. The present *post-hoc* analysis can therefore only provide a first indication that administration of the patch can be continued satisfactorily in the majority of patients undergoing surgery and should be confirmed by additional studies.

Current guidelines recommend the use of oral opioid-containing medications before, during, and after surgery when dopaminergic medication is suspended or slowly being re-established [13]. In case oral administration of opioid-containing medications is not possible, parenteral routes are suggested. The rotigotine transdermal patch avoids invasive routes of administration providing an alternative to patients’ regular oral dopaminergic medication for the perioperative period. Although switching from different dopaminergic medications to the rotigotine patch has not been investigated systematically in RLS patients, overnight switching was effective and well tolerated in patients suffering from PD [29,30]. Switching and re-switching of regular antiparkinsonian medication to the patch was also considered feasible in perioperative PD management [16].

## Conclusions

Although the data were obtained from a study which was not designed to assess rotigotine use in the perioperative setting, this *post-hoc* analysis suggests that treatment with rotigotine transdermal patch can be maintained during the perioperative period in the majority of patients and might thus permit uninterrupted alleviation of RLS symptoms. Rotigotine transdermal patch may be a feasible treatment alternative in perioperative situations where administration of oral treatments is limited, unavailable, or not applicable. Further prospective studies are clearly warranted to confirm this finding.

## Competing interests

BH has been a consultant or acted on advisory boards for GSK, BI, UCB, Lundbeck, Jazz, Nycomed, Sanofi, Pfizer, Merz, Cephalon, and has been a speaker for GSK, BI, UCB, Pfizer, Cephalon,

WHO has received honoraria for consultancy and for serving on scientific advisory boards, and travel support from UCB; and honoraria for consultancy and lecture fees from Teva, Novartis, GlaxoSmithKline, Boehringer Ingelheim, Orion Pharma, and Merck Serono.

ES and LB are employees of UCB Pharma, Monheim, Germany; both receive UCB stock options.

## Authors' contributions

BH participated in study conception, data interpretation, manuscript writing, and manuscript review and critique. WHO participated in data interpretation and critical revision of the manuscript. ES and LB participated in study conception, data analysis and interpretation, and critical revision of the manuscript. All authors read and approved the final manuscript.

## Pre-publication history

The pre-publication history for this paper can be accessed here:

http://www.biomedcentral.com/1471-2377/12/106/prepub
